# The Role of Environmental and Nutritional Factors in the Development of Inflammatory Bowel Diseases: A Case–Control Study

**DOI:** 10.3390/nu16152463

**Published:** 2024-07-29

**Authors:** Victor Serrano-Fernandez, Jose Alberto Laredo-Aguilera, Carlos Navarrete-Tejero, Brigida Molina-Gallego, Angel Lopez-Fernandez-Roldan, Juan Manuel Carmona-Torres

**Affiliations:** 1 Facultad de Fisioterapia y Enfermeria, Universidad de Castilla-La Mancha, Avda. Carlos III s/n, 45071 Toledo, Spain; victor.serrano3@alu.uclm.es (V.S.-F.); carlos.navarrete@uclm.es (C.N.-T.); brigida.molina@uclm.es (B.M.-G.); angel.lopezfernandez@uclm.es (A.L.-F.-R.); juanmanuel.carmona@uclm.es (J.M.C.-T.); 2 Grupo de Investigación Multidisciplinar en Cuidados (IMCU), Universidad de Castilla-La Mancha, Avda. Carlos III s/n, 45071 Toledo, Spain

**Keywords:** inflammatory bowel disease, Crohn’s disease, ulcerative colitis, risk factors, nutrition, health practices

## Abstract

Background: The incidence and prevalence of inflammatory bowel diseases (IBD) are increasing around the world, especially in Western countries. The objective of this study was to evaluate the health habits of healthy controls and individuals with IBDs to identify possible risk factors for IBD development. Methods: A case-control study was conducted among Spanish participants over 18 years of age. A self-administered questionnaire was completed by subjects to collect information on several sociodemographic variables and habits, such as the consumption of tobacco, alcohol, antibiotics, nonsteroidal anti-inflammatory agents and macronutrients; anxiety and depression; and quality of life. Results: The main risk factors identified were age; living in an urban environment; anxiety; and excessive consumption of proteins, carbohydrates and fats. In addition, the consumption of fibre had a preventive effect against IBD development. Conclusions: Age, anxiety and living in urban areas pose a risk of suffering from IBD, as does the excessive consumption of certain macronutrients. However, the consumption of fibre has a protective effect on the development of some IBD types.

## 1. Introduction

IBDs are a group of gastrointestinal diseases, including Crohn’s disease (CD) and ulcerative colitis (UC) [1,2]. IBDs are pathologies involving alternating episodes of clinical activity and phases of inactivity or clinical remission [1,3]. The symptoms of IBDs include fever, diarrhoea, abdominal pain and rectal bleeding, among others [3]. Although the clinical manifestations of CD and UC are similar, there are differences in the distribution of intestinal inflammation between CD and UC [3]. For example, in CD, inflamed intestinal areas are discontinuous, and these areas of inflammation may occur in any part of the digestive tract. In contrast, in UC, the inflammation tends to be continuous and is limited to the colon [3].

IBDs tend to occur in young people, with 25% of patients being diagnosed before 18 years of age [4]. In addition, it is estimated that in areas such as North America and Europe, more than 1.5 and 2 million people, respectively, suffer from IBDs [5]. The total incidence and prevalence of IBDs in Europe is 725 and 28.6 cases per 100,000 inhabitants, respectively [6]. The incidence and prevalence of CD are 20.2 and 322 cases per 100,000 people, respectively, whereas the incidence and prevalence of UC are 24.3 and 505 cases per 100,000 inhabitants, respectively [5].

CD and UC are idiopathic pathologies for which the aetiology is unknown [7]. However, genetic, immunological and environmental factors play fundamental roles in the development of IBDs [7,8]. Among the genetic factors, mutations in 215 genes have been found to increase the risk of developing IBDs [9]. Some loci, such as IL23R, JAK2 or STAT3, are common for both diseases, whereas others are specific to CD (NOD2 and ATG16L1) and UC (IL10, IL22 or IFN-γ) [10,11]. Immunological factors associated with IBDs include alterations in the function of regulatory T cells and pathways involving molecules such as IL-1β or IL-17 [12,13]. With respect to environmental risk factors, the most recent literature emphasises that nutritional habits, exposure to common drugs such as tobacco or alcohol, the use of antibiotics and nonsteroidal anti-inflammatory drugs (NSAIDs) and emotional factors such as anxiety or depression are associated with IBD development [14,15,16,17,18].

The gut microbiome plays a crucial role in the pathophysiology of IBDs [19]. Compared with healthy individuals, IBD patients have a lower diversity of Faecalibacterium prausnitzii and Eubacterium rectale and a lower concentration of metabolites, such as short-chain fatty acids (SCFAs), among others, but a greater diversity of species such as Escherichia coli, Actinomyces, and Ruminococcus, among others [19,20,21].

In terms of environmental factors, it should be noted that the consumption of fibre in the diet has a preventive effect on the development of IBDs in healthy subjects [14]. Additionally, the consumption of fibre in subjects with CD without intestinal stenosis is effective in conjunction with biological therapies at the time of pathological remission [22]. With digestive diseases such as IBD, nutrition should be a fundamental pillar of their prevention and management [14,17,22,23]. The influence of diet could be due to the ability of the diet to modulate the intestinal microbiome, preventing or promoting imbalances between microbial species [24,25].

In contrast, tobacco use influences the risk of developing IBDs [26]. The underlying mechanism by which this phenomenon occurs is unknown; however, the ability of tobacco to alter the microbiome could be directly related to this phenomenon [27]. Interestingly, it should be noted that tobacco use acts as a protective factor against flare-ups in patients with UC [26,28]. Individuals who consume alcohol have been found to have an intestinal microbiome similar to that of IBD patients; therefore, alcohol consumption could be a risk factor for the development of IBDs [29].

Antibiotic use has been associated with IBD development [16,17]. As with the previously described examples, this phenomenon could be due to the alteration of the intestinal microbiome of healthy subjects [21]; this effect is dependent on the antibiotic and the dose consumed by the subjects [17]. In addition, in the case of NSAIDs, alterations in microbial diversity at the intestinal level are known [30]. Notably, because the mechanism of action of NSAIDs involves the inhibition of the enzyme cyclooxygenase 1 (COX-1), NSAID consumption promotes digestive ulcers and has been associated with IBD flare-ups [18].

Among emotional factors, both depression and anxiety are psychiatric disorders associated with IBD development in predisposed populations [31]. This association could be related to the gut–brain–microbiota axis, known as the bidirectional connection of the gut with the central nervous system, thus linking factors such as anxiety and depression to intestinal inflammation [32,33]. The presence of these psychiatric disorders is related to the production of substances such as IL-6, among other inflammatory mediators [34,35,36]. In addition, anxiety can trigger the activation of type 17 T helper (Th17) cells [34].

Regarding the prevention of IBDs, environmental factors have special relevance since they are modifiable through good health habits [14]. As mentioned above, adequate fibre consumption has a protective effect against IBD development. In addition, all the environmental factors described above are connected in that they can all produce imbalances in intestinal microbiota diversity [19,20,21,27,30,32,37]. However, for some habits, such as alcohol consumption, doubts about its possible role in the risk of IBD development persist [38], whereas NSAID use is associated with the occurrence of relapses. Nevertheless, the role of NSAID use in CD and UC development is still uncertain [18]. Psychiatric factors are more common in patients with IBDs, but the role of these factors in the development of these pathologies is not clear [39,40]. Notably, IBDs pose a significant burden on public health in European countries [41,42]. This burden is due to increased cases throughout Europe, especially in countries with greater industrialization [41,43].

Therefore, the objective of this study was to analyse health habits and environmental factor exposures among a group of individuals with IBDs (before diagnosis) and healthy controls to identify possible risk factors for IBD development.

## 2. Materials and Methods

### 2.1. Study Design

A cross-sectional case–control study was carried out according to Strengthening the Reporting of Observation studies in Epidemiology (STROBE) checklist guidelines [44].

### 2.2. Participants and Sample Size

The study sample included Spanish individuals who were older than 18 years, including those with IBDs and healthy controls. The data were collected between June 2023 and January 2024.

Patients with IBDs were included in the case group. Criteria for inclusion in the case group were as follows: (1) a diagnosis of an IBD, (2) disease in clinical remission and (3) age over 18 years. The case group exclusion criterion was the inability to communicate through information and communication technologies (ICTs).

Criteria for inclusion in the control group were (1) healthy status, (2) access to ICTs (3) and age over 18 years. The control group exclusion criterion was a diagnosis of a gastrointestinal pathology other than IBDs.

The GRANMO program (Version 7.12 April 2012) was used to calculate the sample size. In a case-control study carried out in Japan by Sakamoto N et al. in 2005 [45], including 108 patients with UC, 126 patients with CD and 211 controls, a greater risk of UC was associated with carbohydrate and sweetener consumption (odds ratio (OR) = 2.12; 95% CI: 1.08 to 4.17). Therefore, with an accepted alpha value of 0.05 and a beta value of 0.2 for a bilateral research design, for this study, 143 cases and 143 controls were required to detect a minimum OR of 2.12. The rate of exposure in the control group was assumed to be 0.5. In addition, a loss to follow-up rate of 10% was estimated, and the POISSON approach was used.

### 2.3. Variables

Sociodemographic variables: These variables included age, sex, marital status, educational level, profession, social class and place of residence.

Condition of interest: All the subjects who completed the form were asked to indicate whether they had suffered from an IBD. In this way, each participant was categorised as a case or a control depending on whether they suffered from an IBD.

Descriptive variables for cases of IBD: For the group of cases only, information on the type of IBD, need, medication for the disease, and adherence to pharmacological treatment were collected.

Variables related to health habits and nutrient consumption: Data on health habits, the need for medication and emotional disorders were collected 12 months before the diagnosis of IBD in the case group and 12 months before the survey was completed in the control group. Information was collected regarding the daily consumption of calories, carbohydrates, fats, proteins and fibre. In addition, information on alcohol and tobacco consumption (with smoking status based on World Health Organization (WHO) criteria) [46], antibiotic and NSAID use, alcohol dependence, number of cigarettes smoked daily and types of antibiotics and NSAIDs consumed were collected. Self-perceived anxiety and depression were also analysed.

Variables related to quality of life: Data concerning subject-perceived quality of life were collected.

### 2.4. Instruments

The following tools were used for the collection and expression of data for the different study variables.

Alcohol consumption

The Alcohol Use Disorders Identification Test (AUDIT) was used to evaluate alcohol consumption [47]. This questionnaire consists of 10 multiple-choice items. The subjects were classified according to their risk of alcohol addiction based on their AUDIT score. Low, medium, high and probable addiction risk was assigned to subjects with scores of 0–7, 8–15, 16–19 and 20–40, respectively.

Anxiety and depression

The Goldberg Anxiety and Depression Scale was used to evaluate anxiety and depression [48]. This scale is composed of a subscale for anxiety and another for depression. The cut-off scores were four affirmative responses for the anxiety subscale and two for the depression subscale, with higher scores indicating a greater risk of anxiety and depression, respectively.

Macronutrient intake

A Food Frequency Questionnaire (FFQ) was used to calculate differences in the consumption of macronutrients. The values of macronutrients consumed daily were calculated based on participant responses in a manner similar to previous studies [49,50].

Quality of life

The WHO Quality of Life Brief Version (WHOQOL-BREF) questionnaire, which is a simplified version of the 26-item WHOQOL-100 questionnaire, was used to assess the quality of life [51]. This questionnaire does not have cut-off points; higher scores indicate higher subject quality of life. The WHOQOL-BREF is composed of 4 domains (physical, psychological, social and environmental) that are evaluated separately, with a score obtained for each domain and a total score comprising the sum of scores for the 26 items. Items 3, 4 and 26 are reverse-scored.

Adherence to pharmacological treatment

The Morisky–Green questionnaire was used to analyse pharmacological treatment adherence among subjects with IBD [52]. This questionnaire consists of four items. Patient treatment adherence is considered when the responses to the four items are No, Yes, No and No.

### 2.5. Data Collection

A questionnaire was disseminated online. This survey was designed via the Microsoft Forms tool and was accompanied by an information sheet and an informed consent form. Once the online informed consent form was read and signed, the subjects were allowed to continue and complete the survey.

To recruit subjects for the case group, the questionnaire was disseminated throughout the Spanish territory through patient associations such as the Association of Crohn’s Disease and Ulcerative Colitis Patients (ACCU) and its different sections in autonomous communities and provinces, the Asociación Cordobesa de Enfermedad Inflamatoria Intestinal (ACEII) and the Asociación Socio Sanitaria de Enfermedades Inflamatorias Intestinales (ASSEII).

On the other hand, to recruit subjects for the control group, the questionnaire was disseminated via IMCU’s social media across the entire country. Thus, individuals interested in participating could do so by responding to the questions.

### 2.6. Ethical Considerations

The study was approved by the Social Research Ethics Committee (SREC) of the University of Castilla-La Mancha with protocol code CEIS-694878-S2S2, approval date 11 April 2023. All the participants read the information sheet and provided their consent to participate in the study by completing the disseminated survey.

### 2.7. Data Analysis

For the analysis of the data, SPSS version 29, obtained through a UCLM licence, was used. Qualitative variables are presented as counts (n) and percentages (%). Quantitative variables are presented as arithmetic means (m) and standard deviations (SD).

The categorical variables were compared via the chi-square test and are presented in contingency tables. In addition, a Pearson correlation analysis of the scores of the different tools administered in the survey and the nutritional values was performed. Logistic regression analysis was subsequently carried out to identify the possible factors associated with IBD development.

Participants were classified as cases or controls based on whether they suffered from an IBD.

All tests were two-tailed, and results within a 95% confidence interval, assuming an alpha error of *p* < 0.05, were considered significant.

## 3. Results

**Figure 1:**
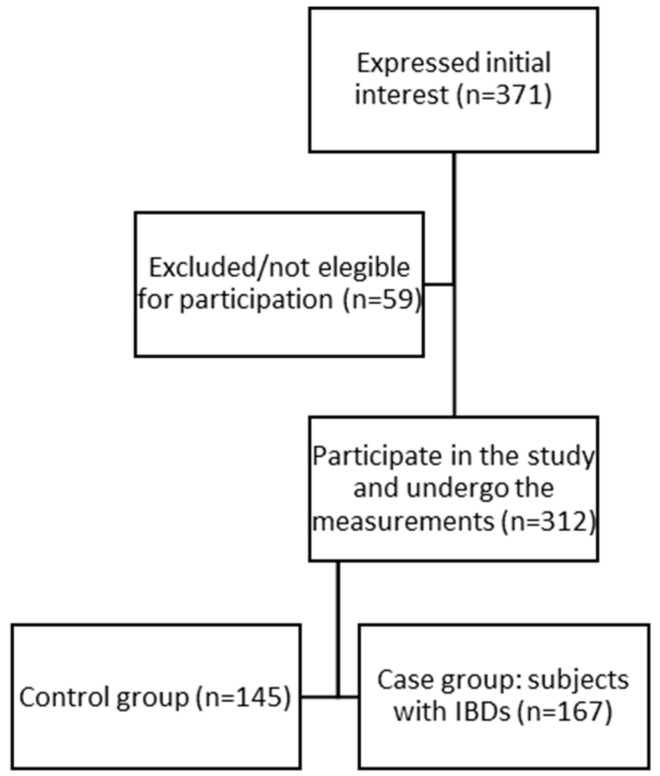
Participant selection flow chart.

Initially, 371 subjects responded to the survey; however, 59 cases were excluded because of errors in completing the questionnaire. Therefore, the final sample included 312 subjects: 167 subjects in the case group of individuals suffering from IBDs (95 with CD and 72 with UC) and 145 subjects in the control group. Figure 1 shows the participant selection process. Most respondents were women (66 vs. 34%), and the mean age of the participants was 41.11 years (SD ± 14.02). Among the total sample, most of the subjects were married (47.8%), had completed university studies (37.8%) and lived in an urban environment (55.1%). Table 1 shows the sociodemographic characteristics of both groups. Furthermore, Table 2 presents the differences in lifestyle among participants.

Among the participants, 53.5% had some type of IBD, whereas 46.5% were healthy controls. Among the subjects with IBDs, 56.9% had CD, and 43.1% had UC. Most of the subjects with IBDs required medication at the time of the study (88%), the most common being biological therapies (53.9%). The descriptive characteristics of the patients with IBDs are presented in Table 3.

### 3.1. Risk Factors for IBD

Regarding risk factors (Table 2), significant between-group differences (*p* < 0.001) were found in terms of tobacco consumption, with 21.6% and 17.2% of cases and controls reporting that they smoked, respectively. In addition, 29.3% of cases were former smokers, whereas 11.8% of controls were former smokers. The mean WHOQOL-BREF score prior to diagnosis was lower in the case group than the control group, with scores of 85.64 (±17.52 SD) and 90.84 (±14.51 SD), respectively (*p* = 0.004).

Significant between-group differences were found for self-perceived anxiety and depression, with 110 subjects with IBD with self-perceived anxiety vs. 58 without self-perceived anxiety (*p* < 0.001) and 110 subjects with IBD with self-perceived depression vs. 73 without self-perceived depression (*p* = 0.005). The mean scores for both subscales were significantly different, with mean values of 4.8 ± 2.99 vs. 3.04 ± 2.76 for the anxiety subscale and 3.61 ± 2.84 vs. 2.16 ± 2.28 for the depression subscale for the case vs. control groups.

In addition, significant differences were also found in terms of the consumption of macronutrients, including calories, carbohydrates and total fats, between the IBD group and healthy subjects (Table 4). Additionally, subjects with IBD consumed lower amounts of fibre prior to diagnosis.

No statistically significant differences between groups were found regarding the use of antibiotics, NSAIDs, alcohol or tobacco. Additionally, no differences were found between subgroups when the different risk factors were analysed. These factors were common for CD and UC patients in the present study.

### 3.2. Descriptive Differences between Subgroups

When each IBD type was analysed separately, several statistically significant differences were found. Among these differences, patients who did not need medication were more likely to have CD than UC (18.9 vs. 2.8%). In addition, the need for aminosalicylates was greater in patients with UC, whereas the use of biological therapies was more common in the subgroup of patients with CD.

On the other hand, adherence to treatment did not significantly differ between subgroups, with 66.3% and 62.5% of patients with CD and patients with UC adhering to treatment, respectively, and 33.7% and 37.5% of patients with CD and patients with UC not adhering to treatment, respectively.

### 3.3. Bivariate Correlations between Risk Factors

Table 5 shows the correlations between the different risk factors for the present study, expressed as the means ± SDs. Positive and significant correlations were found between the anxiety and depression subscale scores and between total calorie consumption and the consumption of various macronutrients.

On the other hand, negative and significant correlations were found between Goldman’s anxiety and depression subscale scores and WHOQOL-BREF scores and the consumption of fibre in the diet, as well as the amount of carbohydrates and fibre consumed in the diet.

### 3.4. Logistic Regression

Table 6 shows the ORs of the factors associated with the development of IBDs. According to the multiple logistic regression model, the statistically significant variables related to IBD development were living in an urban environment (OR = 3.877) and self-perceived anxiety (OR = 3.039).

Similarly, people who were diagnosed with an IBD had greater daily carbohydrate consumption (OR = 1.039, *p* = 0.047) and lower total daily fibre consumption (OR = 0.921, *p* = 0.002).

## 4. Discussion

The results of this research revealed that certain habits (e.g., smoking) and mental disorders (e.g., anxiety and depression) were more common in patients with IBDs prior to their diagnosis than in healthy controls. At the nutritional level, the case group consumed more calories, lipids, carbohydrates, and less fibre than the control group. In addition, living in a rural environment seems to have a preventive role in the development of these pathologies.

A previous study revealed that rural housing was a protective factor against IBDs [53]. Specifically, a case-control study carried out in a paediatric Asian sample [53] found that living in a rural environment was a protective factor against IBDs, whereas, in the present study, an OR of developing an IBD of 3.877 was obtained for living in an urban area. Additionally, differences in the quality of the diet and the cost of food have been reported between urban and rural areas, with the consumption of processed foods, sugar and saturated fats being greater in urban areas and the consumption of wholegrain products and legumes being greater in rural areas [54]. Moreover, a study demonstrated that the consumption of ultra-processed food and oxidative stress were higher in patients with IBD, with a positive and statistically significant correlation between these variables [55]. Another study [56], in contrast to the present study, did not find differences between the quality of the diet in individuals living in the two different environments; however, the study did find differences in nutritional habits due to other factors, such as age, the presence of chronic diseases or living alone. In addition, several studies have shown that fibre consumption is a protective factor against IBDs [45,57]. Specifically, a case-control study [57] found that fibre consumption was a more significant protective factor than the present study. However, another multicentre case-control study [45] revealed that the daily consumption of foods rich in fibre had a protective effect, similar to the results of the present work. The protective effect of fibre against IBD may be due to its role as a substrate for the microbiota, which synthesizes SCFAs that have anti-inflammatory effects and regulate mucosal immunity [19]. Additionally, SCFA levels are lower in individuals with IBD compared to healthy populations [58]. According to the results of the present study and those of another study, the excessive consumption of fats constitutes a risk factor for suffering from IBDs [57]. Protein consumption was found to be a protective factor for IBD development, with an OR of 0.866 [57]. However, the results from the multicentre study mentioned above [45], in accordance with the results of the present study, revealed that consuming foods rich in protein was a risk factor for IBD development. The excessive consumption of carbohydrates was found to be a risk factor for suffering from CD and UC [59], which is consistent with the results of the present study.

Disorders such as anxiety or depression are common in patients with IBDs before their diagnosis [60,61,62]. When comparing the results of the present study with those of other studies [60,62], anxiety was found to be an important risk factor. Depression was not found to be a risk factor in the logistic regression; however, depression was more common in the case group than in the control group in the present study. This finding is in accordance with the results of other investigations [60,61,62,63] in which the presence of depression was also common among subjects with IBDs. Notably, both anxiety and depression may be present before IBD diagnosis and may persist years later [60]. The relationship between these disorders before and after IBD diagnosis could be because both depression and anxiety are related to the production of inflammatory mediators [34,35,36], which are also associated with a sedentary lifestyle and an increased incidence of IBDs [64]. Both anxiety and depression are inversely correlated with physical activity [65,66], which indicates that those individuals with less physical activity have a greater risk of suffering from IBDs, anxiety and depression [64,65,66]. Additionally, a case–control study demonstrated that individuals who engaged in daily physical activity had a lower risk of developing IBD, with an OR of 0.58, which was statistically significant. This finding indicates that physical activity acts as a protective factor against these conditions [67].

The results of the present study revealed that both anxiety and depression were significantly inversely correlated with quality of life, which was also reported in another study [68]. This study additionally found correlations between IBD symptoms and anxiety, depression and quality of life [68]. In the present study, subjects in the case group perceived a lower quality of life than subjects in the control group. Quality of life is often compromised in people already diagnosed with some type of IBD [68,69,70]. However, to our knowledge, no studies have analysed quality of life prior to IBD diagnosis. From the perspective of the present study sample, although the case group comprised patients not yet diagnosed with an IBD, these individuals already presented symptoms of their disease, which may have decreased their quality of life.

The present study also revealed that the rate of antibiotic use in the case group was slightly greater than that in the control group, but the difference was not statistically significant. This result is similar to the results of a previous study [53] in which the number of IBD patients who consumed antibiotics was greater than the number of healthy controls who used antibiotics. Interestingly, the need for antibiotics is increased in other situations, such as gastrointestinal infections, which are also associated with an increased IBD risk [71]. The rate of NSAID use in the present study was lower in the case group; by contrast, a cohort study [72] revealed that those people who consumed NSAIDs more than 15 days a month had an age-adjusted OR of 1.60 for CD and 1.95 for UC. However, the methodology and patient recruitment used in the present study were different from those used in this study, in which a sample of exclusively female nurses was evaluated. In addition, this difference can also be explained by the fact that the consumption of NSAIDs is very widespread among the general population and is more common among women [73].

When comparing data on tobacco use, some observational studies obtained results similar to those of the present study, with tobacco use being a risk factor for IBD development [74,75,76]. In addition, the risk of UC was found to be higher in former smokers [76]. This finding indicates that tobacco use influences the risk of UC development in a healthy population [74,75,76]; however, tobacco use has been found to prevent flare-ups in patients who have already been diagnosed with an IBD [76].

With respect to alcohol consumption, the present study did not find significant differences in alcohol consumption between the case and control groups. This finding coincides with that of a prospective investigation that did not find a relationship between alcohol consumption and the risk of suffering from IBDs [77]. However, another study reported an OR of IBD development of 1.545 for subjects who consumed alcohol [78]. Although alcohol is harmful to the digestive tract [37], in other cases, in the context of suffering from an IBD [29], it is difficult to find associations between alcohol consumption in a healthy population and the risk of developing CD or UC. This lack of an association could be because, like other factors, such as taking NSAIDs, alcohol consumption is a widespread practice in developed countries [79], which makes it difficult to find differences between groups and makes it necessary to recruit a greater number of subjects. Notably, the study that found differences recruited a sample of 458,109 subjects [78], whereas the study that found no differences included 121,701 participants [77].

In addition to the symptoms associated with the presence of IBD and its consequences for the quality of life of the subjects [3,69], the increase in cases of IBDs in industrialised areas is becoming a public health concern in Western countries [41,42,43]. This concern is related to the high demand for pharmacological treatments and health care in Western patients [41,80]. In this context, the prevention of new IBD cases becomes a necessity. Healthcare personnel play a fundamental role in the care and prevention of IBDs; therefore, it is important for these personnel to know the risk factors associated with the development of these pathologies.

### Limitations and Strengths

Among the limitations of the present study was that the questionnaire used for data collection was self-administered. The subject-reported consumption of calories and macronutrients might not reflect their real consumption. Because the main objective of the study was to observe the differences between case and control groups, the complete FFQ was not used. We found that fibre consumption is a preventive factor for IBD; however, this study did not analyse the differences between various types of fibre (fermentable vs. non-fermentable), so it would be interesting to investigate this in future studies. Additionally, since the questionnaire was administered through patient associations, it was not possible to match cases with controls. Since cases described their habits before diagnosis, recall difficulties may introduce bias. The habits of the cases may have changed with the onset of symptoms before diagnosis. To mitigate this confounding factor, we asked cases about their habits from one year prior to diagnosis and employed a standardized questionnaire consistent with practices in previous articles [81,82]. Furthermore, owing to the cross-sectional case–control design of the present investigation, it is not possible to determine the direction of the associations obtained between the different variables.

As strengths, it should be noted that a sufficient sample size was recruited to ensure the validity and reliability of the results, and the questionnaire was completed by individuals throughout the national territory. Another strength of this study is the use of a standardized questionnaire that incorporated validated tools. Furthermore, because subjects with IBDs had to answer the questionnaire from their own perspective before being diagnosed with an IBD, the risk factors studied and reflected in this study were not modified by the presence of these pathologies.

## 5. Conclusions

Among the factors studied, age, anxiety and living in an urban environment pose a risk of suffering from IBDs, as does the excessive consumption of certain macronutrients (fats, proteins and carbohydrates), whereas the consumption of fibre seems to have a protective effect on the development of CD or UC. Additionally, although no significant differences were found, a greater proportion of people with IBDs were smokers and more subjects with IBDs used antibiotics.

Given that IBDs begin in the digestive system, proper nutrition should be one of the fundamental pillars of both prevention and care in managing these diseases. In addition, it is important to pay attention to mental health since anxiety was found to be a risk factor for IBD development and to be correlated with depression, considering that the presence of anxiety can lead to depression and vice versa. In addition, both anxiety and depression were correlated with a lower quality of life, with this quality of life being lower in patients with IBDs than in healthy subjects. Therefore, in addition to focusing on nutritional factors when preventing IBDs in a healthy population, other factors, such as psychiatric factors and the environment in which the individual lives, must be considered. Knowing these high-risk factors for IBDs is essential for health policy-makers so that they can take these factors into consideration when developing prevention and awareness campaigns.

## Figures and Tables

**Table 1:** Sociodemographic characteristics.

Variable	IBD		*p* Value
	Yes *n* (%)	No *n* (%)	
Age (mean years ± SD)	45.07 ± 13.13	36.54 ± 13.66	<0.001
Sex			0.512
Male	54 (32.3)	52 (35.9)	
Female	113 (67.7)	93 (64.1)	
Marital status			0.022
Single	63 (37.7)	80 (55.2)	
Married	92 (55.1)	57 (39.3)	
Divorced	10 (6)	7 (4.8)	
Widower	2 (1.2)	1 (0.7)	
Education level			0.267
None	1 (0.7)	1 (0.7)	
Basic studies	9 (5.4)	14 (9.7)	
Secondary studies	68 (40.7)	46 (31.7)	
University studies	89 (53.2)	84 (57.9)	
Profession			<0.001
Health personnel	42 (25.1)	57 (39.3)	
Administration	63 (37.7)	34 (23.4)	
General services	34 (20.4)	25 (17.2)	
Pensioner	18 (10.8)	4 (2.8)	
Student	7 (4.2)	21 (14.5)	
Unemployed	3 (1.8)	4 (2.8)	
Social class			0.018
Class 1	17 (10.2)	14 (9.7)	
Class 2	21 (12.6)	15 (10.3)	
Class 3	27 (16.2)	35 (24.1)	
Class 4	54 (32.3)	26 (17.9)	
Class 5	36 (21.6)	48 (33.1)	
Class 6	12 (7.1)	7 (4.9)	

IBD—inflammatory bowel disease.

**Table 2:** Differences in Lifestyle.

Variable	IBD		*p* Value
	Yes *n* (%)	No *n* (%)	
Living environment			<0.001
Urban environment	113 (67.7)	59 (40.7)	
Rural environment	54 (32.3)	86 (59.3)	
Smoker			<0.001
Yes	36 (21.6)	25 (17.2)	
No	82 (49.1)	103 (71)	
Former smoker	49 (29.3)	17 (11.8)	
Number of cigarettes smoked daily			0.778
Less than 5	20 (23.5)	8 (19)	
Between 6 and 15	41 (48.2)	20 (47.6)	
More than 16	24 (28.3)	14 (33.4)	
Alcohol consumption			0.463
Yes	22 (13.2)	26 (17.9)	
No	140 (83.8)	116 (80)	
Former drinker	5 (3)	3 (2.1)	
AUDIT score (mean ± SD)	2.75 ± 3.41	3.55 ± 4.19	0.063
Risk of alcohol addiction			0.423
Low risk	151 (90.4)	127 (87.6)	
Medium or high risk	16 (9.6)	18 (12.4)	
WHOQOL-BREF questionnaire score (mean ± SD)	85.64 ± 17.52	90.84 ± 14.51	0.004
Use of antibiotics			0.072
Yes	52 (31.1)	32 (22.1)	
No	115 (68.9)	113 (77.9)	
Type of antibiotic			0.129
Penicillins	13 (25)	13 (40.6)	
Cephalosporins	0 (0)	1 (3.1)	
Macrolides	0 (0)	3 (9.4)	
Quinolones	4 (7.7)	1 (3.1)	
Sulfonamides	1 (1.9)	0 (0)	
Glycopeptides	0 (0)	1 (3.1)	
Rifamycins	1 (1.9)	1 (3.1)	
Nitroimidazoles	3 (5.8)	1 (3.1)	
Phosphomycins	6 (11.5)	3 (9.4)	
Not specified	24 (46.2)	8 (25.1)	
Use of NSAIDs			0.098
Yes	54 (32.3)	60 (41.4)	
No	113 (67.7)	85 (58.6)	
Type of NSAIDs			0.022
Enantyum	5 (9.1)	16 (27.1)	
Ibuprofen	17 (30.9)	22 (37.3)	
Diclofenac	1 (1.8)	0 (0)	
Naproxen	10 (18.2)	7 (11.9)	
COXIBs	5 (9.1)	0 (0)	
Not specified	17 (30.9)	14 (23.7)	
Goldberg anxiety subscale score (mean ± SD)	4.8 ± 2.99	3.04 ± 2.76	<0.001
Anxiety			<0.001
Yes	110 (65.9)	58 (40)	
No	57 (34.1)	87 (60)	
Goldberg depression subscale score (mean ± SD)	3.61 ± 2.84	2.16 ± 2.28	<0.001
Depression			0.005
Yes	110 (65.9)	73 (50.3)	
No	57 (34.1)	72 (49.7)	

IBD—inflammatory bowel disease; AUDIT—Alcohol Use Disorders Identification Test; SD—standard deviation; WHOQOL-BREF—World Health Organization Quality of Life Brief Version; NSAIDs—nonsteroidal anti-inflammatory drugs; COXIBs—cyclooxygenase-2 inhibitors.

**Table 3:** Characteristics of the IBD patients according to IBD type.

Variable	Type of IBD		*p* Value
	Crohn‘s Disease *n* (%)	Ulcerative Colitis *n* (%)	
Age (mean years ± SD)	43.98 ± 13.47	46.51 ± 12.63	0.218
Sex			0.566
Man	29 (30.5)	25 (34.7)	
Female	66 (69.5)	47 (65.3)	
IBD medication			0.001
Yes	77 (81.1)	70 (97.2)	
No	18 (18.9)	2 (2.8)	
Type of IBD medication			<0.001
Aminosalicylates	5 (5.3)	34 (47.2)	
Immunosuppressants	11 (11.6)	7 (9.7)	
Biological therapies	61 (64.2)	29 (40.3)	
Without medication	18 (18.9)	2 (2.8)	
Adherence to treatment			0.609
Yes	63 (66.3)	45 (62.5)	
No	32 (33.7)	27 (37.5)	

IBD—inflammatory bowel disease.

**Table 4:** Macronutrient consumption comparison according to the presence of IBDs.

Variable	Presence of IBDs		*p* Value
	Yes m (±SD)	No m (±SD)	
Total calories	912.75 (±420.06)	770.51 (±348.33)	<0.001
Total proteins	30.28 (±13.39)	26.04 (±12.87)	0.005
Total lipids	21.75 (±19.1)	14.1 (±14.39)	<0.001
Total carbohydrates	158.16 (±67.94)	142.26 (±62.66)	0.033
Fibre	16.6 (±11.95)	19.05 (±11.54)	0.068

IBD—inflammatory bowel disease.

**Table 5:** Bivariate correlations.

Simple Correlation										
	Age	AUDIT Score	Goldberg Anxiety Score	Goldberg Depression Score	WHOQOL-BREF Total Quality of Life Score	Daily Kcal	Daily Protein	Daily Fats	Daily Carbohydrates	Daily Fibre
Age	-	−0.087	0.060	0.043	−0.160 **	0.107	0.006	0.075	0.096	0.011
AUDIT score		-	0.093	0.094	0.051	0.007	0.089	0.154 **	−0.036	−0.096
Goldberg anxiety score			-	0.755 **	−0.435 **	−0.017	0.034	0.062	−0.048	−0.124 *
Goldberg depression score				-	−0.501 **	−0.018	0.003	0.050	−0.031	−0.113 *
WHOQOL-BREF total quality of life score					-	−0.050	−0.030	−0.068	−0.036	0.062
Daily kcal						-	0.819 **	0.582 **	0.917 **	0.445 **
Daily protein							-	0.608 **	0.697 **	0.312 **
Daily fats								-	0.292 **	−0.196 **
Daily carbohydrates									-	0.679 **
Daily fibre										-

Data are presented as Pearson’s correlation coefficients. * *p* < 0.05, ** *p* < 0.001. AUDIT—Alcohol Use Disorders Identification Test; WHOQOL-BREF—World Health Organization Quality of Life Brief Version.

**Table 6:** Logistic regression model for the associations between inflammatory bowel diseases and the sociodemographic and health characteristics of the Spanish sample.

Variable	Simple Logistic Regression		Multiple Logistic Regression *	
	Crude OR (95% CI)	*p* Value	Adjusted OR (95% CI)	*p* Value
Age	1.047 (1.029–1.066)	<0.001	1.048 (1.027–1.070)	<0.001
Living environment				
Urban environment	3.050 (1.919–4.849)	<0.001	3.877 (2.223–6.732)	<0.001
Rural environment	Reference		Reference	
Anxiety				
Yes	2.895 (1.825–4.591)	<0.001	3.039 (1.772–5.211)	<0.001
No	Reference		Reference	
Total daily calories	1.001 (1–1.002)	0.002	0.993 (0.985–1.001)	0.085
Total daily protein	1.027 (1.008–1.046)	0.006	1.048 (1.000–1.097)	0.050
Total daily lipids	1.034 (1.015–1.053)	<0.001	1.059 (0.998–1.123)	0.057
Total daily carbohydrates	1.004 (1–1.007)	0.035	1.039 (1.000–1.078)	0.047
Total daily fibre	0.982 (0.964–1.001)	0.069	0.922 (0.875–0.971)	0.002

CI—confidence interval; OR—odds ratio; * R2 Nagelkerke value for multiple logistic regression = 0.361.

## Data Availability

All datasets employed and/or analysed in this study are available from the corresponding author upon reasonable request.
